# Metal Ions in Biological Systems, Vol. 34: “Mercury and its Effects on Environment and Biology”

**DOI:** 10.1155/MBD.1997.287

**Published:** 1997


					BOOK REVIEW
Volume 34 of the series METAL IONS IN BIOLOGICAL SYSTEMS'
"Mercury and its Effects on Environment and Biology", ed. Astrid $igel
and Helmut Sigel (648 pages, $ 250.00, Marcel Dekker)
Volume 34 of the series METAL IONS IN BIOLOGICAL SYSTEMS, entitled
"Mercury and its Effects on Environment and Biology", covers many up-
to date multidisciplinary research lines in this area.
Speciation of Hg(ll) and CH3Hg(II) compounds and its applications to the
determination of mercury in lakes, rivers and marine environment, including
boreal surface waters, are the topics of chapters 1 to 4.
Mercury pollution in soil, including the biogeochemistry of Hg in the air-soli-plant
system, as well as its bioaccumulation in the food chain, are covered by the six
next chapters.
Physiology, toxicology, metabolism and transport of mercury are discussed in
chapters 11 to 13.
The last six chapters deal with the effects of Hg on the immune system, the impact
of this metal released from dental "silver" fittings on antibiotic resistance, the
inhibition of brain tubulin-GTP interactions by mercury, the reaction of Hg(ll) with
nucleic acids and the use of 2D 1H{99Hg} HMQC NMR, Hg-responsive gene
regulation and bacterial Hg-resistance, several research lines that were initiated
only quite recently and that are clearly aimed to stimulate future work in this area.
This book gives an excellent up-to-date coverage of the field and will be very
useful to people working in this area of research.
287

				

